# Gastrointestinal and Urinary Tract Endometriosis: A Review on the Commonest Locations of Extrapelvic Endometriosis

**DOI:** 10.1155/2018/3461209

**Published:** 2018-09-26

**Authors:** Dimitra Charatsi, Ourania Koukoura, Irontianta Gkorezi Ntavela, Foteini Chintziou, Georgia Gkorila, Manthos Tsagkoulis, Themistoklis Mikos, George Pistofidis, Jiannis Hajiioannou, Alexandros Daponte

**Affiliations:** ^1^Department of Obstetrics and Gynecology, University Hospital of Larissa, Thessaly, Larissa, Greece; ^2^1st Department of Obstetrics & Gynecology, Aristotle University of Thessaloniki, Papagheorgiou General Hospital, Thessaloniki, Greece; ^3^Department of Gynaecologic Endoscopic Surgery, Lefkos Stavros Hospital of Athens, Athens, Greece; ^4^University of Thessaly, Medical Faculty, Larissa, Greece

## Abstract

Extrapelvic endometriosis is a rare entity that presents serious challenges to researchers and clinicians. Endometriotic lesions have been reported in every part of the female human body and in some instances in males. Organs that are close to the uterus are more often affected than distant locations. Extrapelvic endometriosis affects a slightly older population of women than pelvic endometriosis. This might lead to the assumption that it takes several years for pelvic endometriosis to “metastasize” outside the pelvis. All current theories of the pathophysiology of endometriosis apply to some extent to the different types of extrapelvic endometriosis. The gastrointestinal tract is the most common location of extrapelvic endometriosis with the urinary system being the second one. However, since sigmoid colon, rectum, and bladder are pelvic organs, extragenital pelvic endometriosis may be a more suitable definition for endometriotic implants related to these organs than extrapelvic endometriosis. The sigmoid colon is the most commonly involved, followed by the rectum, ileum, appendix, and caecum. Most lesions are confined in the serosal layer; however, deeper lesion can alter bowel function and cause symptoms. Bladder and ureteral involvement are the most common sites concerning the urinary system. Unfortunately, ureteral endometriosis is often asymptomatic leading to silent obstructive uropathy and renal failure. Surgical excision of the endometriotic tissue is the ideal treatment for all types of extrapelvic endometriosis. Adjunctive treatment might be useful in selected cases.

## 1. Introduction

Pelvic endometriosis usually refers to lesions proximal to the uterus such us the ovaries, the fallopian tubes, the uterine ligaments, and the surrounding pelvic peritoneum. Extrapelvic endometriosis on the other hand, is affecting other areas of the body, including the vagina, vulva, cervix and perineum, the urinary system, the gastrointestinal tract, the thoracic cavity including lung and pleura, extremities, skin, and central nervous system. Nevertheless, the term of extragenital pelvic endometriosis describes in a more accurate way endometriotic lesions involving pelvic organs such as rectum, sigmoid, and bladder. Diagnosis and treatment of extrapelvic endometriosis is complex due to the variety of affected sites, the lack of accurate diagnostic methods, and the management of the disease by different specialties.

### 1.1. Epidemiology

Extrapelvic endometriosis is a fairly rare phenomenon. The exact prevalence is basically unknown due to the small number of well-designed epidemiological studies. The incidence of the disease depends on the population studied, methods used to make the diagnosis, and the expertise of the surgeon. Data derive mostly from case series and case reports that describe endometriotic lesions in virtually every part of the female body and in some cases in the male body. There are no reports however of endometriotic disease in the heart or spleen [1]. In general endometriosis affects 5–10% of women of child-bearing age, but only a small proportion of these women are diagnosed as having the extrapelvic type of the disease [2, 3]. Extrapelvic endometriosis is generally diagnosed in a slightly older population than pelvic endometriosis. The median age at time of diagnosis is 34–40 years, whereas pelvic endometriosis is commonly diagnosed a decade earlier [4]. The frequency of the disease decreases while the distance to the uterus increases [1].

### 1.2. Pathogenesis—Classification

Endometriosis is an enigmatic disease, also known as “the disease of theories.” Similar to pelvic endometriosis, no clear consensus exists on which theory best explains the pathogenesis of the different types and locations of extrapelvic endometriosis. Traditional theories include metaplastic transformation of the pelvic peritoneum (coelomic metaplasia) [1]; transplantation of endometrial tissue either through retrograde menstruation, iatrogenic relocation during surgical procedures, or by hematogenous or lymphatic spread [2]; and induction of undifferentiated mesenchyma tissue, through unknown mediators in the shed endometrium, to form endometriotic lesions in extrauterine regions [3]. There is no uniformly accepted staging system for extrapelvic endometriosis. Markham et al. published a classification system which divides extrapelvic lesions into four classes: Class I: endometriosis that involves the gastrointestinal tract; Class U: endometriosis involving the urinary tract; Class L: endometriosis involving the lungs and thorax; and Class O: Endometriosis involving all other sites. A further staging includes the classification of the lesions based on the exact location and size of the defect [1].

## 2. Gastrointestinal Tract Endometriosis

### 2.1. Epidemiology

**Figure 1:**
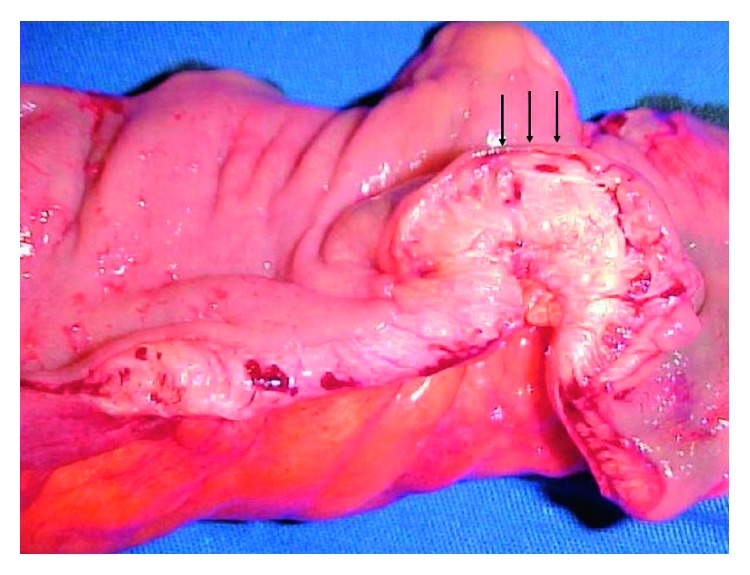
Macroscopic appearance of endometriotic nodule of the sigmoid colon. Arrows indicate the intact mucosal layer [11].

**Figure 2:**
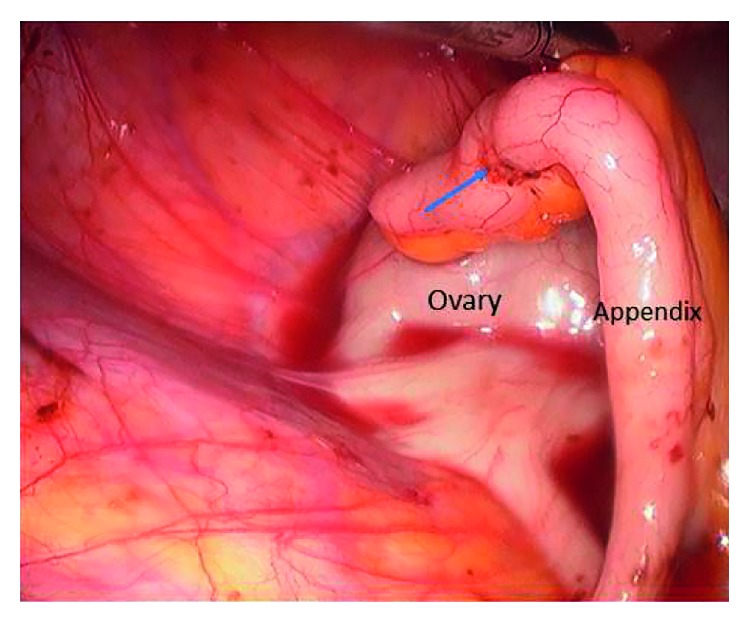
Appendiceal endometriosis. The appendix is attached to the right ovary. The arrow indicates the endometriotic infiltration of the appendix [13].

**Figure 3:**
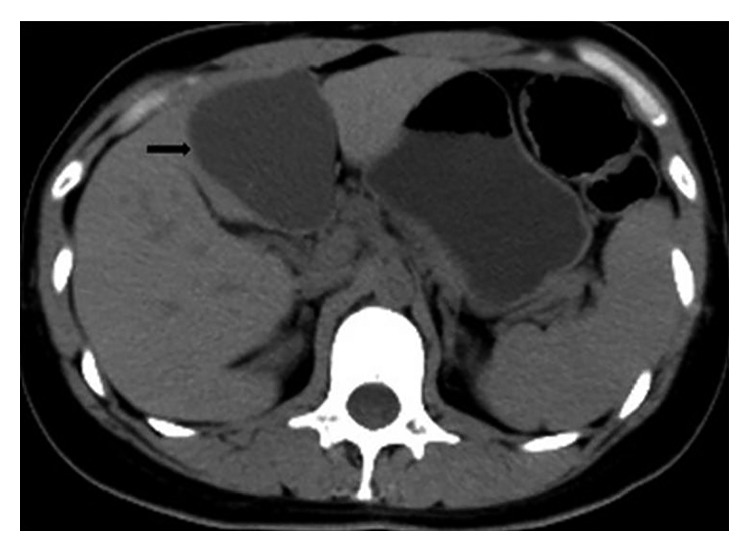
Computed tomography demonstrating a 6.5 × 6 cm endometriotic lesion in the left hepatic lobe.

The gastrointestinal tract is the most common location of extrapelvic endometriosis (and extragenital pelvic endometriosis when referring to rectum, sigmoid, and bladder) [5–7]. Gastrointestinal involvement is reported in up to 3.8–37% of women diagnosed with endometriosis [8]. Adolescent women, women of reproductive age, as well as menopausal women may be affected [9]. The sigmoid colon is most commonly involved, followed by the rectum, ileum, appendix, and caecum [10]. The rectum and the sigmoid are the most common locations in 95% of the patients (Figure 1) [11, 12]. Appendiceal endometriosis is found in 5 to 20% of patients (Figure 2) [13–17]. Small intestine lesions mostly involve the terminal ileum and account for up to 5–16% of gastrointestinal endometriosis cases [18–20]. Extremely rare locations that have been reported include the gallbladder, the Meckel diverticulum, stomach, and endometriotic cysts of the pancreas and liver. Twenty-one cases of cystic liver masses were diagnosed as hepatic endometriomas Figure 3 [21].

### 2.2. Pathogenesis

The high incidence of endometriotic lesions in gastrointestinal sites close to the uterus supports the theory of implantation due to retrograde menstruation. Endometrial tissue is implanted through the fallopian tubes to the intestine, given the proximity of the two organs. Superficial endometriotic implants confined in the serosal layer of the colon are usually asymptomatic. Conversely, deep infiltrating lesions may provoke severe gastrointestinal symptoms [22]. Remorgida and colleagues suggested a system for staging gastrointestinal tract endometriosis which best correlated with patients' symptoms [23]. The bowel specimen affected by endometriosis can be histologically classified into four stages: stage 0, when the endometriotic tissue is only affecting the peritoneum and the subserosal connective tissue (not reaching the subserous plexus); stage 1, endometriotic foci are located in the subserous fat tissue or adjacent to the neurovascular branches (subserous plexus), rarely involving the external muscle layer; stage 2, the muscular wall and the Auerbach plexus are involved; and stage 3, the infiltration is reaching the submucosal nervous plexus or the mucosa [23].

Most of the endometriotic lesions of the gastrointestinal tract are confined to the serosal layer and surrounding connective tissue (stage 0). This type of lesions is not considered as deep endometriotic disease by many researchers [24]. According to this, deep gastrointestinal endometriosis can be made only when invasion of the muscularis layer is established [25, 26]. Deeper lesions are uncommon, however, with only few reports of endometriosis penetrating the bowel's lumen [27].

### 2.3. Symptoms

**Figure 4:**
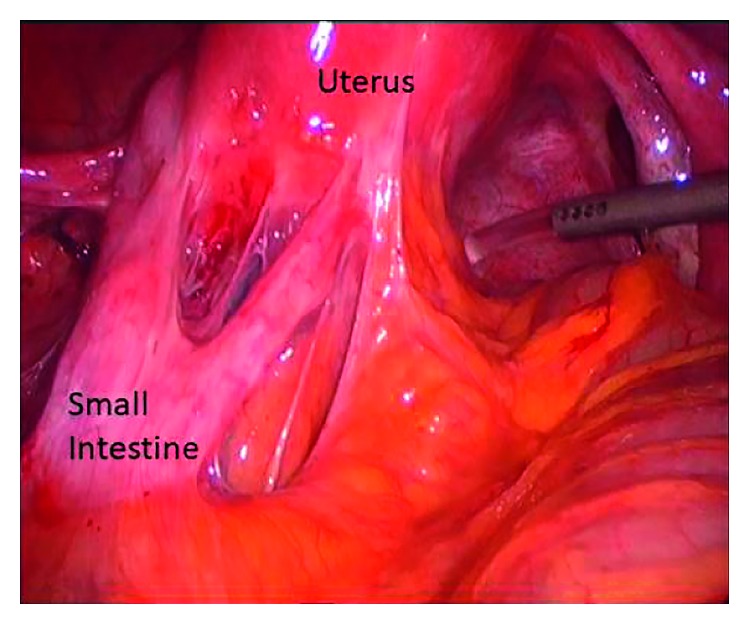
Dense adhesions of the small bowel with the uterus, in a patient with severe infiltrating endometriosis.

Most cases of gastrointestinal endometriosis are asymptomatic [7]. When present, symptoms of intestinal endometriosis depend on the location of the disease and the depth of the invasion. When the lesions are confined in the serosal layer, symptoms are similar to those of women with pelvic endometriosis [23]. These include dysmenorrhea, dyspareunia, and infertility [28]. Other symptoms are usually present when a sclerosing and fibrotic reaction in the bowel wall causes kinking or narrowing of the bowel lumen, leading to distension or stretching during bowel movements (Figure 4). Similar to pelvic endometriosis, the severity of symptoms does not always correspond to the extent of the disease [25, 26].

A solid preoperative diagnosis is difficult to be established, since many of the symptoms can mimic a wide spectrum of diseases, including irritable bowel syndrome, infectious diseases, ischemic colitis, inflammatory bowel disease, ileocolonic intussusception, appendicitis, and malignancy [29–31]. Symptoms, in general, include crampy abdominal pain, dyschezia, tenesmus, meteorism, constipation, melena, diarrhea, vomiting, hematochezia, pain on defecation, and after meals. The traditional cyclical pattern of symptomatology has not been confirmed by recent studies which postulate a rather noncyclical chronic pelvic pain as a more persistent symptom [32]. Cyclical symptoms that aggravate during menses, however, have also been reported in a small number of patients [33, 34]. Since intestinal mucosa is rarely affected, rectal bleeding is also an unusual symptom, reported in 0 to 15% to 30% of patients [15, 35, 36]. Bleeding can also occur due to severe bowel obstruction and ischemia [32, 37]. Acute bowel obstruction due to stenosis is a scarce complication reported only in cases when severe small bowel involvement is present or in the presence of dense pelvic adhesions [38]. Likewise, perforation of the affected bowel represents an extremely rare entity that has been reported in pregnant and nonpregnant women [39, 40]. Sporadic cases of appendiceal rupture and intussusception have also been reported [41, 42].

### 2.4. Diagnosis

**Figure 5:**
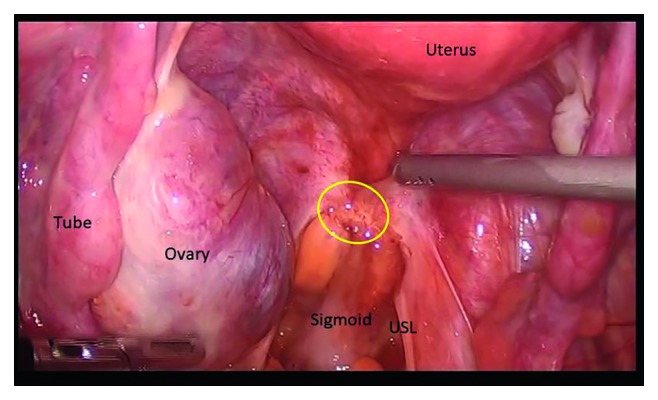
Infiltrating endometriotic lesion in the rectovaginal septum involving the sigmoid and the uterosacral ligament.

General examination is rarely helpful in differentiating intestinal endometriosis from other intestinal disorders or from pelvic endometriosis. In fact, a lot of women with bowel endometriosis have been treated for irritable bowel syndrome before a final diagnosis can be made [43]. Recognition often requires a high index of suspicion and a careful history with a timed physical exam prior to menstruation. Accuracy of diagnosis depends on the imaging technique used, the location and size of the lesion, and also the expertise of the observer. Digital examination of the vagina or rectum may detect a hard, tender nodule either on the posterior vaginal fornix, or the rectal wall which is indicative of bowel involvement (Figure 5). Radiologic studies are often being performed because of the nonspecific character of the patients' symptoms and signs. However, there are no radiologic or diagnostic findings that are specific for endometriosis [44].

**Figure 6:**
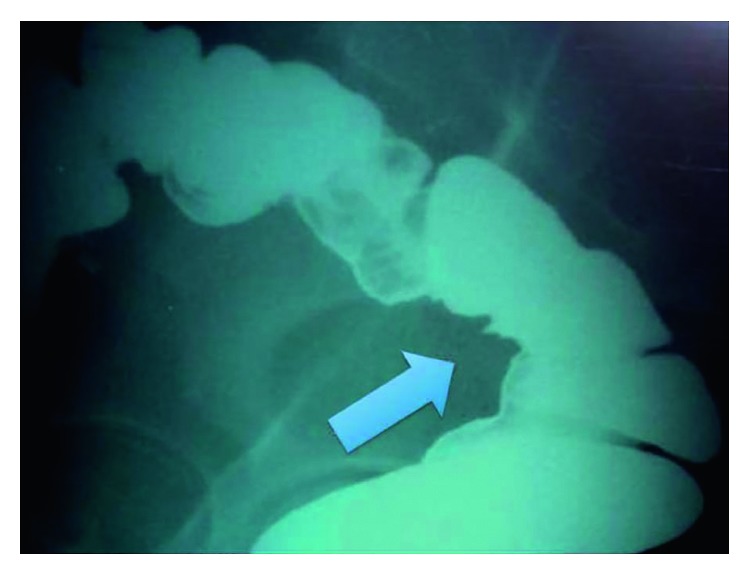
Barium enema demonstrating (arrow) an extrinsic bowel compression in a patient with intestinal endometriosis [11].

**Figure 7:**
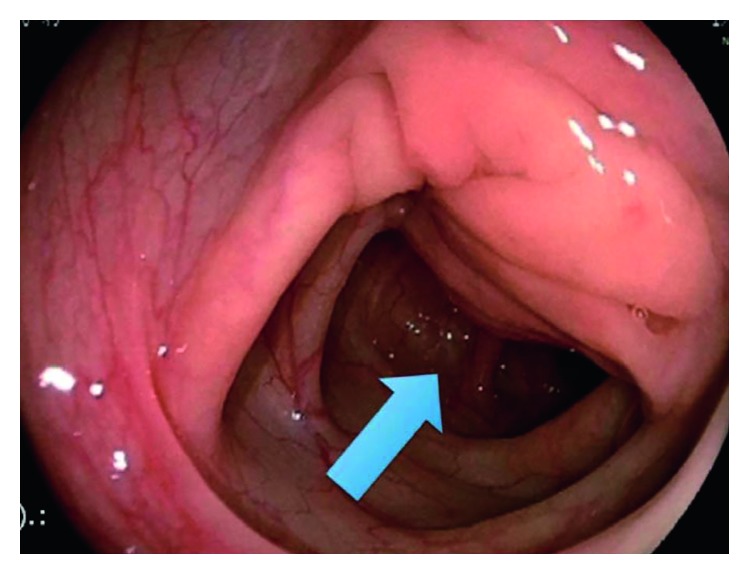
Colonoscopy in a patient with sigmoid endometriosis. The arrow indicates an indentation of the lumen of the bowel with no mucosal involvement [11].

A recent analysis demonstrated that, so far, no imaging modality is accurate enough to overall detect endometriosis compared to surgery [45]. Ultrasound imaging is of some value but there are a few reports in the literature for the use of vaginal and endorectal ultrasound in the diagnosis of submucosal lesions of the rectosigmoid. Sensitivity and specificity vary extremely in different studies [46–48]. By means of transvaginal ultrasound, a deep endometriotic lesion of the colon is usually detected in the anterior wall of the rectosigmoid. The typical image is that of a hypoechoic irregular mass which often involves the left uterosacral ligament [49]. Biscaldi et al. reported the usefulness of multislice CT combined with distention of the colon by rectal enteroclysis for intestinal endometriosis [50]. Traditional CT, however, although valuable in evaluation of pelvic endometriomas has limited use in the diagnosis of the intestinal form of the disease. MRI is considered the most useful examination for bowel endometriosis; however, there are not enough data regarding its utility in diagnosing and monitoring endometriotic lesions in the bowel [2, 51]. Barium enema might demonstrate an extrinsic bowel compression, stenosis, or filling defect, and enteroclysis is the preferred method of investigation for small bowel involvement (Figure 6) [11, 52]. Colonoscopy is helpful to exclude malignancy; however, superficial intestinal endometriotic lesions cannot be seen on proctoscopy, rectoscopy, or colonoscopy. The endoscopic appearance of an endometriotic implant is not diagnostic even when mucosal involvement is present (Figure 7) [11, 53]. Endoscopic biopsy may be helpful, particularly during an episode of bleeding, but the biopsy must be deep enough to establish diagnosis.

**Figure 8:**
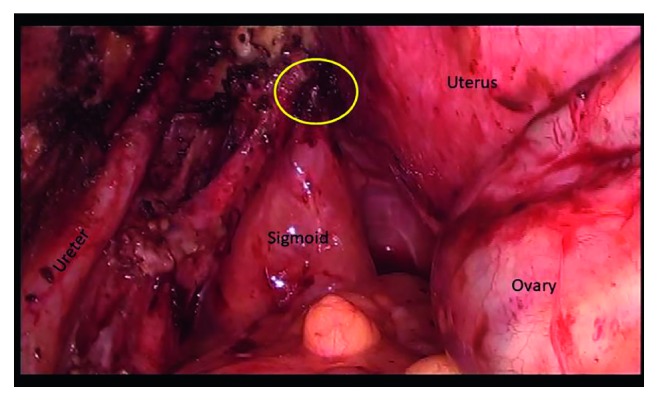
Laparoscopic view of an endometriotic nodule of the sigmoid attached to the left uterosacral ligament.

Primarily laparoscopy or laparotomy with histopathological confirmation of endometriosis remains the gold standard for the diagnosis of gastrointestinal endometriosis (Figure 8). Accurate preoperative diagnosis is very difficult, and most cases are found accidentally at surgery [31]. Even when laparotomy is performed, it depends on the skill of the surgeon in recognizing the endometriotic sites of the gastrointestinal system.

### 2.5. Treatment

**Figure 9:**
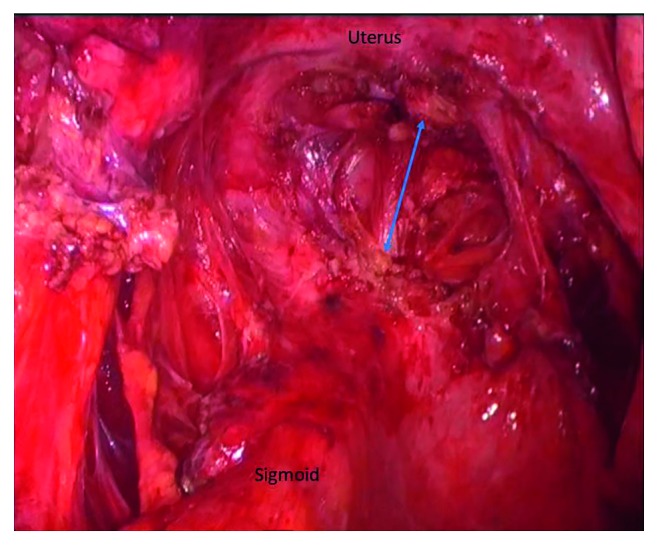
Superficial resection of an endometriotic nodule of the sigmoid.

There is not enough data to support the most effective therapeutic modality for gastrointestinal endometriosis. The treatment should always be individualized, depending on the patient's age and desire to maintain fertility, the presence and severity of symptoms, and also the location of the disease. Treatment options include surgery or hormonal agents, although in most cases surgical resection of the endometriotic lesions is the only option for long-term disease remission (Figure 9).

The medications used in the treatment of endometriosis are danazol, progestins, oral contraceptive pills, gonadotrophine-releasing hormone (GnRH) agonists, and mifepristone (RU-486). Each has been successful on a limited basis [33, 54, 55]. Although medical treatment is offered with the aim of relieving symptoms, it is not definitely curative since pain symptoms recur at discontinuation of treatment [56]. Noteworthy, medication can be given pre- or/and postoperatively or if the patient is unsuitable for surgery [57]. The aim of postoperative medical treatment is symptoms relief and probable reduction of recurrences on the grounds of the hypothesis that “adjuvant” hormonal therapy against microscopic lesions may act like chemotherapy for malignancies; nonetheless, available clinical evidence denies this approach which actually is not surprising taking under consideration that hormonal therapy causes temporary suppression of endometriotic cells' action in contradiction to chemotherapic agents which destroy cancer cells of micrometastases [56, 58]. However, long-term postsurgical hormonal therapy can be offered for the prevention of ovarian endometriomas' recurrences and dysmenorrhea but not for other pain symptoms [59, 60]. In particular, Seracchiolli et al observed that continuous long-term postoperative administration of oral contraceptive pills is more effective in reducing recurrent dysmenorrhea related to endometriosis compared with cyclic administration; anyway, the choice of the regimen depends on a woman's preference concerning her menstruation [55]. Moreover, evidence suggests a beneficial effect of a levonorgestrel-releasing IUD on the prevention of dysmenorrhea, which may facilitate women's long-term adherence to the treatment [55, 61].

Researchers have developed novel medical agents in order to overcome side effects associated with the common hormonal treatments of endometriosis. Dienogest, aromatase inhibitor (AI), GnRH antagonists, antitumour necrosis factor-*α* (TNF-*α*), and selective estrogen or progesterone receptor modulators (SERMs and SPRMs) are several new therapeutic options; dienogest, AI, and GnRH antagonists are effective medicines with good tolerance and safety, while the results for SERMs and SPRMs are highly controversial and anti-TNF-*α* is in the animal testing stage [62]. Additionally, Harada et al. suggest that dienogest can be a new conservative approach for extragenital endometriosis-related pain and in particular for rectosigmoidal and bladder endometriosis [63]. Noteworthy, evidence exists in favour of postoperative treatment with dienogest aiming to prevention of recurrence and pain relief, while administrated immediately after recurrence and for long-term use, dienogest is a better option than GnRH analogues [64, 65]. Last but not least, dienogest might help in maintaining fertility in patients with endometriosis by avoiding the damage of repeat surgeries to ovarian reserve [64].

As far as bowel endometriosis is concerned, similar to pelvic endometriosis, hormonal therapy may improve symptoms but does not prevent the progression of the disease [8]. Moreover, most hormonal treatments prevent conception and may have a considerable risk of side effects; therefore, long-term administration is not feasible. The side effects and limitations of each therapy need to be discussed meticulously with patient [8]. However, studies have shown that hormonal suppression improves pain and gastrointestinal symptoms in women whose degree of bowel stenosis is <60% [66].

Surgery is the choice of treatment for intestinal endometriosis when there are symptoms as intestinal obstruction, bleeding, and severe pain and if malignancy is suspected. Surgical procedures include segmental full, deep-partial, or superficial-thickness rectal resection, depending on the extent and depth of bowel infiltration [67]; it is estimated that deep endometriosis invading the bowel occurs in 8–12% of women with endometriosis. The number and the size of intestinal deep endometriosis lesions, the extent of bowel circumference involvement, the depth of the lesions, the distance to the anal verge, and lymphatic dissemination are all crucial parameters to determine the best surgical approach [25]. Surgical resection of the affected bowel seems necessary only in cases of complete obstruction and suspicion of malignancy and unmanageable pain [44]. The absence of gastrointestinal symptoms appears to be predictive of the absence of clinically significant intestinal endometriosis, and bowel resection is not indicated in the asymptomatic patient [7]. Despite the fact that most surgeons favour laparoscopy, experience and skills of the surgeon influence the success. Laparoscopic treatment of colorectal endometriosis, even in advanced stages, has been proven feasible and effective in nearly all patients [68, 69]. Although there are reports that advocate that total abdominal hysterectomy and bilateral salpingo-oophorectomy at the time of bowel resection correlates with improved outcome, this form of treatment is not well established [70]. This procedure should be considered only in perimenopausal women and women who do not desire fertility [44]. To conclude, management of intestinal endometriosis requires multidisciplinary approach and follow-up by a team involving gynaecologists, general surgeons, and gastroenterologists.

## 3. Urinary Tract Endometriosis

### 3.1. Epidemiology

The second most common site of extrapelvic endometriosis involves the urinary system [71]. Endometriosis has been estimated to affect the urinary tract in approximately 0.3 to 12% of cases [72–77]. Bladder and ureteral involvement are the most common sites, with the former representing 80–90% and the latter concerning up to 50% of cases with deep infiltrating endometriosis and 92% of colorectal endometriosis [78, 79]. Renal and urethral endometriosis are extremely rare entities, with an incidence of 4% and 14%, respectively [80, 81]. Women with urinary tract endometriosis are usually on their 30's or 40's and half of them have had prior pelvic surgery [82]. There are several reports of vesical endometriosis arising after a caesarean section [83, 84]. Estrogen replacement therapy has been implicated in increasing the likelihood of developing urinary tract endometriosis even in women with no prior history of endometriosis [85].

### 3.2. Pathogenesis

**Figure 10:**
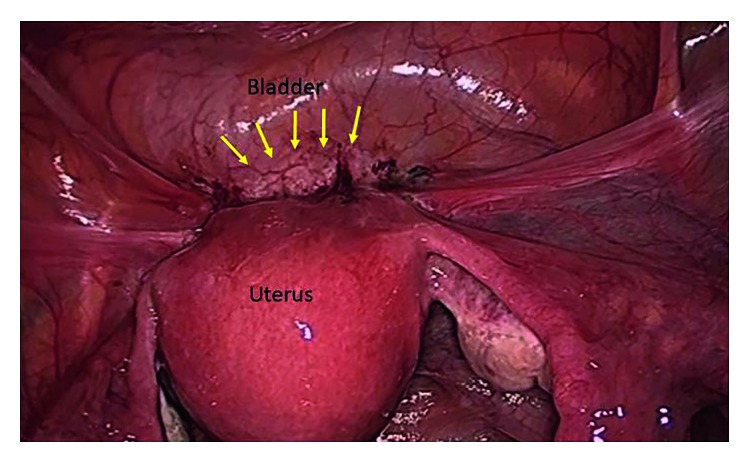
Laparoscopic view of endometriotic lesion. Arrows mark the lesion [13].

The bladder is the most common site, and the lesions are often located in close proximity to the uterus (Figure 10) [13]. Endometriotic lesions of the bladder affect mainly the detrusor muscle in the bladder trigone and bladder apex [76, 86]. The pathogenesis of vesical endometriosis is much debated. The intraperitoneal origin of the disease suggests that that deep infiltrating endometriotic lesions of the bladder result from an intraperitoneal process, which commences with transplantation [76] of ectopic endometrium onto the bladder peritoneum followed by infiltration into the bladder muscularis [87, 88]. Other theories propose that bladder endometriosis could be considered as a bladder adenomyosis as the consequence of a metaplasia of müllerian rests [89] or as result from the extension of adenomyotic lesions of the anterior uterine wall to the bladder [90].

**Figure 11:**
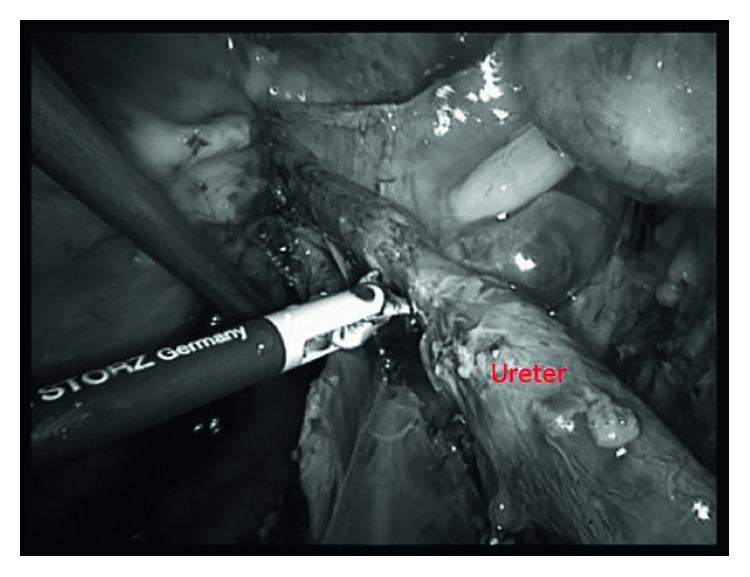
Ureteral stenosis in a patient with endometriosis [91].

Ureteral endometriosis usually is found in the distal third of the ureter below the pelvic brim, and lesions are more common on the left ureter than the right (Figure 11) [91–93]. According to Vercellini et al., this fact may be attributed to the presence of the sigmoid on the left side which creates favorable conditions for endometrial cell seeding from the uterine cavity [92]. Bilateral manifestation of ureters is rare, occurring in 10–20% of patients [76, 94]. Lesions are classified as either extrinsic or intrinsic. The extrinsic form (70–80% of cases) affects the external ureteral tunics through adherence to the surrounding structures or organs, and the intrinsic form (20–30% of cases) invades muscular layer or the ureteral mucosa, sometimes with an intraluminal projection [76, 95].

### 3.3. Symptoms

Diagnosis of vesical endometriosis is difficult, leading to delay in treatment of approximately 4.5 years [78]. Vesical endometriosis is usually presented with suprapubic and back pain or with irritative voiding symptoms [96]. These symptoms generally occur on a cyclic basis and are exaggerated during menstruation. Less than 20% of patients however report cyclical menstrual hematuria, which is considered a pathognomic sign for bladder endometriosis [97–99]. Bladder detrusor endometriosis symptoms may cause symptoms similar to painful bladder syndrome; therefore, diagnosis of bladder endometriosis should be considered in patients with recurrent dysuria and suprapubic pain [100].

Clinical symptoms of ureteral endometriosis are often silent [76, 101, 102]. Since the extrinsic form of the disease is more common resulting from endometriosis affecting the rectovaginal septum or uterosacral ligaments and surrounding tissues, patients present with dyspareunia, dysmenorrhea, and pelvic pain [103]. Abdominal pain is the predominant symptom, occurring in 45% of symptomatic patients [93, 104–106]. Symptoms are often cyclical when the ureter is involved, and cyclic microscopic hematuria is a hallmark of intrinsic ureteral disease [95, 107, 108]. There is a limited correlation between severity of symptoms and the degree of obstruction of the ureter. High degree of obstruction may proceed for a long time without symptoms, leading to deterioration of renal function [76]. Unfortunately, ureteral endometriosis is often asymptomatic leading to silent obstructive uropathy and renal failure [109].

### 3.4. Diagnosis

**Figure 12:**
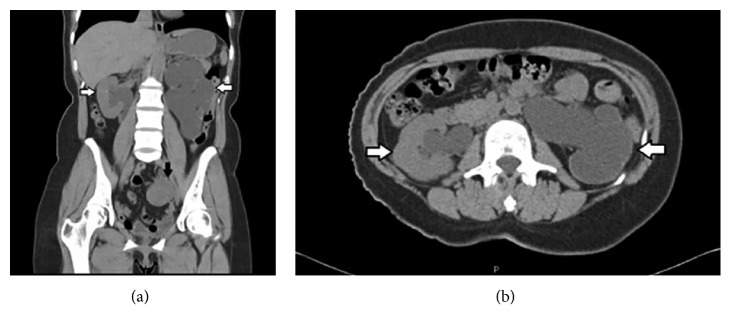
(a) Computed tomography showing a 4.5 cm sized left endometriotic cyst (black arrow) causing ureteral obstruction with severe left hydroureteronephrosis. Enlarged kidneys, with complete loss of left renal parenchyma (white arrows). (b) Milder right hydroureteronephrosis (white arrows) [110].

Physical examination may be suggestive of pelvic endometriosis including pelvic tenderness, adnexal masses, and nodularity of the uterosacral ligaments, although these findings may be absent in patients with vesical endometriosis [107]. A tender pelvic mass in the anterior vaginal fornix is the most common finding on physical examination occurring in one half of patients; however, it does not confirm the diagnosis [82]. Clinical findings or ureteral endometriosis are often silent and this corresponds by the high rate of kidney loss (23–47%) reported by many authors (Figure 12) [103–110]. Therefore, careful evaluation of the uterosacral ligaments and the rectovaginal septum is essential because the presence of such lesions may indicate extrinsic obstruction of the ureter or kidney [78, 111].

**Figure 13:**
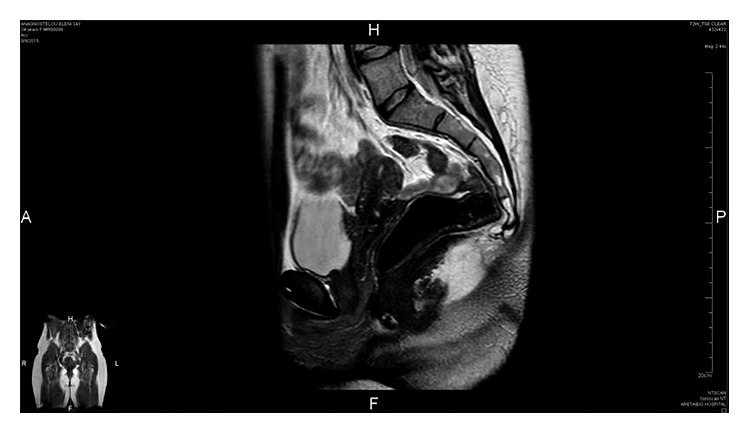
Magnetic resonance image showing a case of deep endometriosis infiltrating the dome of the bladder.

Radiographic imaging studies are widely used but proven to be inadequate in diagnosing definitely urinary tract endometriosis. Ultrasound has been used in order to detect bladder or renal masses. Localized bladder wall thickening, with occasional protrusion into the bladder lumen, represents the main diagnostic criterion [95]. Two-dimensional endoluminal sonography of the ureter may demonstrate the periureteral anatomy, as well as define lesions within the ureteral wall [112]. MRI has advantages over transvaginal ultrasound in diagnosing small endometriotic lesions. The accuracy of MRI in the diagnosis of vesical endometriosis has been reported to be 98% (Figure 13) [113]. Computed tomography or MRI can be helpful in defining the extent of disease before surgery.

**Figure 14:**
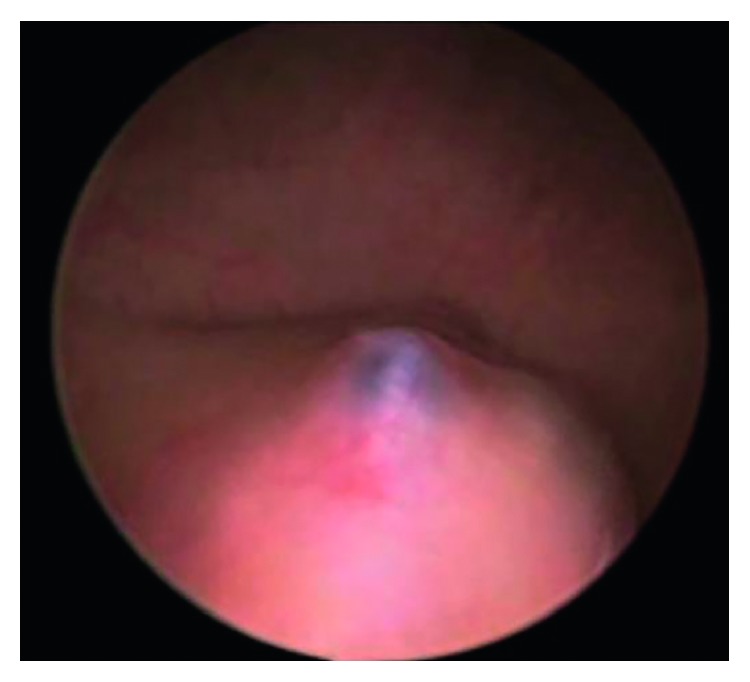
Cystoscopic image of a bluish submucosal endometriotic lesion [13].

Intravenous pyelography (IVP) is widely used as a diagnostic tool in patients with urinary tract symptoms. In cases where vesical endometriosis is present, IVP is usually unremarkable. Intravenous pyelography can identify ureteral obstruction and confirm renal function; however, findings are often nonspecific because the majority of ureteral obstructions are caused by extrinsic disease [114]. Cystoscopy can be valuable in evaluating bladder endometriosis, and biopsy of the suspected areas can provide a definite diagnosis. Bladder endometriomas appear on cystoscopy as edematous bluish submucosal multilocular lesions usually located on the bladder dome or at the bladder base (Figure 14) [13]. The diagnosis should be confirmed by histology, though obtaining a sufficient biopsy may be limited by the submucosal location of the lesion [115]. Even a thorough diagnostic evaluation cannot make an exact diagnosis. Many cases of bladder endometriosis and the majority of cases of ureteral endometriosis are definitely diagnosed during laparotomy or laparoscopy [116]. Laparoscopy is helpful in reaching the diagnosis and gathering information regarding the extent, the location, and size of the lesions.

### 3.5. Treatment

In many instances, therapy of pelvic endometriosis coincides with that of urinary tract endometriosis. However, additional therapeutic goals include elimination or urinary tract symptoms and relief of existing obstruction which might cause renal failure. Many factors must be considered before choosing the right therapeutic approach of urinary tract endometriosis. Choice of treatment depends on patient age, fertility desire, extent of bladder disease, severity of lower urinary tract symptoms, presence of other pelvic disease, and degree of menstrual dysfunction [72]. Preservation of the kidney function is the primary goal, but treatment must be individualized.

Therapy includes both medical and surgical options. All the hormonal agents used to suppress pelvic endometriosis have been also used for the urinary type of the disease with various results [1]. GnRH analogues, danazol, progestins, and estrogen/progestin combination all have had some success in symptomatic relief; however, their use is limited in urinary tract endometriosis especially when there is extensive pelvic disease [96, 103, 115, 117]. Therefore, medical hormone suppression should be considered as an adjuvant therapy to surgery and as preventive therapy for relapses when total hysterectomy with bilateral adnexectomy is not performed or when residual disease is left following surgery [55–57].

Surgical treatment is indicated for patient suffering from symptomatic bladder or ureteral endometriosis. Isolated bladder lesions are mostly treated with local excision or partial cystectomy performed by either laparoscopy or laparotomy [26, 108, 118]. The initial step of treatment when ureteral involvement is present may include ureterolysis. Nezhat et al. presented experience with robot-assisted laparoscopy in treatment of one patient with bladder endometriosis and two patients with urethral endometriosis. The authors prove that this therapy can be a feasible and safe option in women suffering from urinary tract endometriosis [119]. If renal function can be restored, and there is limited ureteral involvement, then ureterolysis is the preferred treatment [73, 111]. Successful ureteroscopic management of intrinsic ureteral disease has also been reported [120, 121]. In case of persistent or recurrent endometriosis, a ureteral resection would be justified. Segmentary ureterectomy termino-terminal anastomosis or ureteral reimplantation into the bladder is performed in cases of intrinsic ureteral disease or extensive ureteral obstruction [93]. The issue of hysterectomy with bilateral salpingo-oophorectomy is an option for patients who do not want to preserve fertility.

## 4. Conclusions

Extrapelvic endometriosis is a rare phenomenon. Most cases of extrapelvic endometriosis are presented to specialties other than gynaecologists. Areas that are close to the uterus are more likely affected by the disease (e.g., bladder and colon) than more distant locations. Both gastrointestinal and urinary tract endometriosis diagnosis is often delayed due to the atypical and nonspecific symptoms. There is a wide spectrum of imaging findings depending on lesion location, morphology, and organ involvement. Diagnosis requires a high degree of suspicion while no accurate diagnostic modality exists that would justify widespread use. Medical history of recurrent symptoms related to the menstrual cycle and imaging abnormalities suggesting the presence of chronic blood products should help in making a correct diagnosis. Histology remains the cornerstone of diagnosis. Surgical treatment is preferable in most cases since all the known medical regimens provide short-term symptomatic relief. Advances in surgical techniques allow a more definite treatment of the disease, although the systematic nature of endometriosis warrants the need for adjunctive treatment in selected cases where radical surgery is not an option.
